# Nigeria, COVID-19 and the dearth of health workers

**DOI:** 10.7189/jogh.10.020379

**Published:** 2020-12

**Authors:** Oluwafemi Temitayo Oyadiran, Luther Agbonyegbeni Agaga, Yusuff Adebayo Adebisi, Don Eliseo Lucero-Prisno

**Affiliations:** 1 Lagos State University College of Medicine, Nigeria; 2 Faculty of Pharmacy, University of Ibadan, Ibadan, Nigeria; 3 Department of Global Health and Development, London School of Hygiene and Tropical Medicine, London, UK

The COVID-19 pandemic continues to sweep the globe, infecting millions of people and causing hundreds of thousands of deaths and massive economic disruption. Africa has so far been largely spared the kind of impact that has caused chaos in developed nations like the United States, Spain and Italy. As of April 28, there were about 34 915 confirmed cases on the African continent [1]. However, this number is expected to keep climbing as Africa is deemed to be experiencing its early stages of the pandemic with her 1.33 billion people at tremendous risk. Being a low-income country in sub-Saharan Africa, Nigeria’s approach to mitigating the impact of the pandemic is of global interest. With a 2020 budget of 10.59 trillion naira and 24.9% of revenues for this budget coming from oil sources and to further expound on the munificent gestures vested on the Nigerian health care, the sector enjoys only 4.14% of the 2020 national budget [2].

The Nigerian health sector has been plagued with inadequacies for decades leaving the upper and middle-class largely distrustful of the system and mostly reliant on medical tourism. There is also paucity of medical professionals secondary to unremitting brain drain. The NMA president said about 75 000 Nigerian doctors were registered with the body, but over 33 000 had left the country, leaving behind only about 42 000 to man all health institutions in the country. The president also noted that, “in rural areas, we have one doctor to 22 000 people, while in towns and cities, we have one doctor to 10 000 Nigerians or one doctor to 12 000 Nigerians, whereas the World Health Organization (WHO) said for any country to have a balanced ratio, it must have one doctor to 600 persons” [3].

Nigeria is a nation where doctors courageously manage all forms of infectious diseases as part of their routine daily activities, including the current COVID-19, but are only guaranteed a monthly hazard allowance of N5000 (US$ 14, Central Bank of Nigeria exchange rate of N361 as of 28 April 2020), with the minister of health not even being aware of such a fee and in the advent of illness or death of a medical doctor while on the job, they are entitled to nothing in health insurance coverage. Whereas, counterparts in a neighboring country, Ghana, will be paid US$4322 as health insurance coverage in the occurrence of illness/death of a doctor in the fight against COVID-19. Health insurance is a necessary incentive, even for a medical doctor, not just because it connotes the importance the health care managers confer on the health workers, but also because it serves as an assurance of protection in the event of the unprecedented.

Prior to the index case on the 27 February 2020, there has been capacity building in epidemic preparedness and the Nigerian Centre for Disease Control (NCDC) had set up 23 public health emergency operations, and four testing centers sparsely distributed in the country with the North lacking a testing facility. As of 30 March 2020, Nigeria had only 350 Intensive Care Unit (ICU) beds to serve her over 200 million populations as estimated by the Nigeria Centre for Disease Control, that is, one in every 570 000 people would have access to critical treatment, using medical practice for the whole population [4]. COVID-19 being a respiratory disease is often complicated by acute respiratory distress syndrome (ARDS), and in such a case, access to intensive care would prove to be lifesaving. But in such a grossly deprived health care system as in Nigeria, only mass hysteria awaits as the number of confirmed cases of COVID-19 continues to skyrocket with alarming possibilities of cases requiring such advanced health services.

In the battle against the COVID-19 pandemic, Nigeria like the rest of the world, is greatly lacking of the needed personal protective equipment (PPE), which is an essential component in the management of this highly infectious disease. So far, she has enjoyed generous PPE donations from well-meaning countries, organizations and individuals and these have contributed to a large extent in the fight against this common global enemy. However, she still desperately needs continued influx of PPE to ensure that health care workers are well protected in rendering their selfless service to humanity. A recent innovation, and by far a development in the right direction, in this regard is the indigenous production of PPE such as facemasks and medical gowns by local textile companies. An initiative well indicative of the resourcefulness of Nigerians in a time of disaster.

Lagos state in conjunction with private organizations have been able to construct isolation centers fitted with ICU while some states converted the orientation camps of the national youth service corps to temporary isolation centers alongside its general hospitals. In order to minimize the toll of the pandemic on citizens, the Lagos state government announced free services for pregnant women and people with health emergencies. The hazard allowance of health workers in Lagos witnessed an increase from US$13 to US$65 in an attempt to incentivize them. Toll free COVID-19 lines for reporting suspected cases were created by the Lagos state government but this has been plagued by hoax calls which have constituted majority of the calls made to the lines. In order to ramp up testing, the NCDC plans to begin testing suspected COVID-19 cases in HIV/Tb laboratories by May 2020 by repurposing at least one Gene Xpert laboratory in each state for COVID-19 testing in addition to its current 12 testing facilities distributed in 8 states in the country. The Lagos State government, which has the highest burden of the disease in the nation, has also set up 20 sampling centers across all local government and private companies have also donated drive-through sample collection booths.

**Figure:**
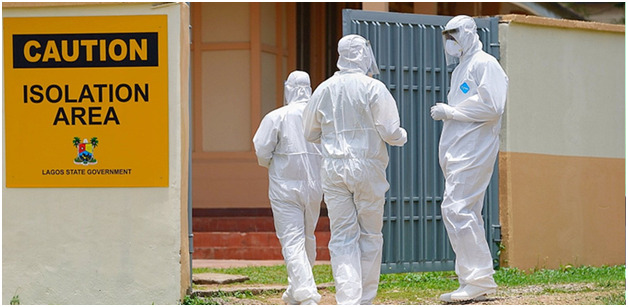
Photo: Healthcare workers in front on a COVID-19 isolation centre in Lagosrom. From: https://guardian.ng/news/covid-19-lagos-discharges-47-nigerians-9-foreigners/ (copyright free).

The imposed lockdown in the nation, in a bid to curb the pandemic, is a laudable effort from the government as similar actions have helped to control the pandemic spread in some other countries such as China, Italy and Spain. However, considering the realities of the densely populated and low-income country, the provision of palliatives to the indigent and generation of a robust economic stimulus package remains a vital responsibility of the government to ensure total compliance.

The untoward economic effect of this pandemic cannot be overemphasized, with oil currently trading at US$21.23 per barrel as at 29 April 2020 [5], the lowest in 18 years, and the diversion of existing funds to combat this menace, the post COVID-19 era of Nigeria appears gloomy.
